# Supplementary material for: Lung Subregion Partitioning by Incremental Dose Intervals Improves Omics-Based Prediction for Acute Radiation Pneumonitis in Non-Small-Cell Lung Cancer Patients
Source: Cancers (Basel). 2022 Oct 6;14(19):4889. doi: 10.3390/cancers14194889 (PMC9564373; doi:10.3390/cancers14194889)
Supplement: Supplementary file 1 [file cancers-14-04889-s001.zip › cancers-1823070-supplementary.pdf]

<u>SUPPLEMENTARY MATERIALS</u>

<u>Contents</u>

Feature Information

<u>SUPPLEMENTARY TABLES</u>

Table S1: The selected features for the three feature groups.

Table S2: The standard deviation of the model performance for all evaluation metrics. The deep green color means a lower value of the evaluated metric. On the contrary, the deep red color represents a higher evaluation value.

Table S3 The previous studies for predicting the ARP using single or multiple omics features for lung cancer patients treated with RT.

<u>SUPPLEMENTARY FIGURES</u>

Figure S1: Mean receiver operating characteristic (ROC) curve of six models using subregion features and the whole lung features.

Supplementary Tables

Table S1. *The selected features for the three feature groups.*

Name	Features
WL-RDF	WL_original_gldm_DependenceVariance_20_binCount<br>WL_log-sigma-6-0-mm-3D_glszm_SmallAreaHighGrayLevelEmphasis_20_binCount<br>WL_log-sigma-1-0-mm-3D_gldm_DependenceNonUniformityNormalized_20_binCount<br>WL_wavelet-HLH_firstorder_Maximum<br>WL_dose_original_glrlm_RunEntropy<br>WL_log-sigma-3-0-mm-3D_glszm_SmallAreaHighGrayLevelEmphasis_20_binCount<br>WL_log-sigma-6-0-mm-3D_glszm_SizeZoneNonUniformityNormalized_20_binCount<br>WL_log-sigma-6-0-mm-3D_gldm_SmallDependenceHighGrayLevelEmphasis_20_binCount<br>WL_wavelet-HLH_glszm_ZoneEntropy_50_binCount<br>WL_wavelet-HLH_glrlm_GrayLevelVariance_20_binCount<br>WL_log-sigma-6-0-mm-3D_glcm_Correlation_100_binCount<br>WL_wavelet-LLH_glszm_GrayLevelNonUniformity_20_binCount<br>WL_wavelet-HHH_glcm_Correlation_20_binCount<br>WL_wavelet-HLH_glszm_ZoneEntropy_100_binCount<br>WL_dose_original_firstorder_Energy<br>WL_wavelet-LHL_firstorder_Skewness<br>WL_original_glcm_ClusterProminence_20_binCount<br>WL_log-sigma-1-0-mm-3D_glrlm_ShortRunHighGrayLevelEmphasis_20_binCount<br>WL_dose_moment_2_3_3<br>WL_log-sigma-1-0-mm-3D_glrlm_GrayLevelVariance_20_binCount<br>WL_wavelet-LLH_glcm_ClusterShade_200_binCount<br>WL_wavelet-HHL_glrlm_ShortRunLowGrayLevelEmphasis_20_binCount<br>WL_wavelet-LLL_glrlm_RunLengthNonUniformityNormalized_20_binCount<br>WL_wavelet-LLH_glszm_ZoneEntropy_20_binCount<br>WL_wavelet-HHH_firstorder_Mean<br>WL_log-sigma-6-0-mm-3D_firstorder_Kurtosis<br>WL_wavelet-LHL_gldm_LargeDependenceHighGrayLevelEmphasis_20_binCount<br>WL_wavelet-HHH_ngtdm_Busyness_50_binCount<br>WL_wavelet-HHH_glcm_InverseVariance_100_binCount<br>WL_wavelet-LHL_glrlm_LowGrayLevelRunEmphasis_20_binCount<br>WL_log-sigma-6-0-mm-3D_glszm_GrayLevelVariance_200_binCount
SR-RDF	SR_10_20_dose_moment_3_0_2<br>SR_0_10_dose_original_glrlm_RunEntropy<br>SR_30_40_log-sigma-6-0-mm-3D_glcm_ClusterProminence_150_binCount<br>SR_30_40_wavelet-HHH_glcm_InverseVariance_50_binCount<br>SR_40_50_log-sigma-1-0-mm-3D_glcm_InverseVariance_20_binCount<br>SR_0_10_dose_original_firstorder_Energy<br>SR_40_50_log-sigma-3-0-mm-3D_firstorder_Variance<br>SR_30_40_dose_original_firstorder_Minimum<br>SR_20_30_dose_moment_3_0_1<br>SR_30_40_wavelet-HLL_firstorder_90Percentile

SR_30_40_log-sigma-3-0-mm-3D_firstorder_InterquartileRange
SR_30_40_wavelet-HLL_ngtdm_Complexity_200_binCount
SR_30_40_maximum_dose
SR_20_30_wavelet-HHH_glrlm_LongRunHighGrayLevelEmphasis_150_binCount
SR_0_10_dose_moment_3_2_0
SR_40_50_dose_moment_1_2_2
SR_20_30_wavelet-LHH_glrlm_LongRunLowGrayLevelEmphasis_20_binCount
SR_20_30_wavelet-HLH_glszm_GrayLevelNonUniformityNormalized_20_binCount
SR_10_20_dose_moment_2_0_0
SR_40_50_dose_original_gldm_DependenceNonUniformity
SR_40_50_wavelet-LLL_glszm_LargeAreaHighGrayLevelEmphasis_50_binCount
SR_10_20_wavelet-LHL_glcm_ClusterProminence_100_binCount
SR_40_50_wavelet-LHH_glcm_Correlation_20_binCount
SR_10_20_wavelet-HHH_firstorder_Kurtosis
SR_40_50_wavelet-HLL_glrlm_RunEntropy_20_binCount
SR_30_40_wavelet-HHL_firstorder_Minimum
SR_10_20_dose_moment_2_1_2
SR_10_20_wavelet-LLL_glcm_Imc2_50_binCount
SR_10_20_log-sigma-1-0-mm-3D_glcm_Imc1_20_binCount
SR_40_50_dose_original_glszm_GrayLevelNonUniformity
SR_20_30_wavelet-HLH_glcm_MaximumProbability_20_binCount
SR_30_40_log-sigma-3-0-mm-3D_ngtdm_Contrast_20_binCount

Abbreviations: glszm: Gray Level Size Zone Matrix, gldm: Gray Level Dependence Matrix, glcm: Gray Level Co-occurrence Matrix, glrlm: Gray Level Run Length Matrix, MCC: Maximal Correlation Coefficient,

Table S2. *The standard deviation of the model performance for all evaluation metrics. The deep green color means a lower value of the evaluated metric. On the contrary, the deep red color represents a higher evaluation value.*

	Cohort	WL-DF	WL-RF	WL-RDF	SR-DF	SR-RF	SR-RDF
AUC	Train	0.03	0.03	0.02	0.03	0.02	0.01
	Test	0.06	0.07	0.04	0.08	0.05	0.05
Acc	Train	0.04	0.05	0.03	0.03	0.03	0.02
	Test	0.07	0.06	0.05	0.08	0.05	0.05
Pre	Train	0.04	0.06	0.05	0.04	0.05	0.05
	Test	0.06	0.08	0.09	0.11	0.08	0.10
Re	Train	0.05	0.06	0.05	0.06	0.06	0.03
	Test	0.13	0.14	0.13	0.12	0.13	0.11
F1	Train	0.04	0.06	0.04	0.05	0.04	0.03
	Test	0.06	0.07	0.07	0.09	0.09	0.08

Colour scale: 0.1 (top) to 0 (bottom)

Abbreviation: Acc: Accuracy; Pre: Precision; Re: Recall.

Table S3. *The previous studies for predicting the ARP using single or multiple omics features for lung cancer patients treated with RT.*

Reference	Features (n)	Classification	Methods	AUC	Patient information
[26]	Radiomics (9)	ARP grade≥2	Logistics regression	0.75	SBRT for 40 stages I NSCLC patients
[24]	Radiomics (8), DDF (5)	ARP grade≥ 3	LASSO	0.68	IMRT/3DCRT for 192 NSCLC patients
[25]	DDF (5), Clinical factors (13), Cytokines (30),	ARP grade≥2	RF, SVM, MLP	0.831	RT for 106 NSCLC patients

	miRNAs (62), SNPs (60)				
27	DDF (11), Clinical factors (21)	ARP grade≥2	RF	0.66	RT for 203 stage II–III NSCLC patients
8	Radiomics (TL-GTV)<br><br>Multi-ROIs radiomics	ARP grade≥2	SVM	0.71<br>0.94	VMAT for 79 stages I-IV Lung cancer patients
9	Radiomics, Dosiomics, Clinical factors	ARP grade≥2	RF	0.771 (V_{20})<br><br>0.763 (V_5)	RT for 701 NSCLC patients
28	Radiomics (486)	ARP grade≥2	Logistic regression	0.871 (Training)<br>0.756 (Testing)	SBRT For 275 stage I NSCLC patients
10	Dosiomics	ARP grade ≥2	LightGBM	0.846	SBRT for 685 NSCLC patients

Supplementary Figures

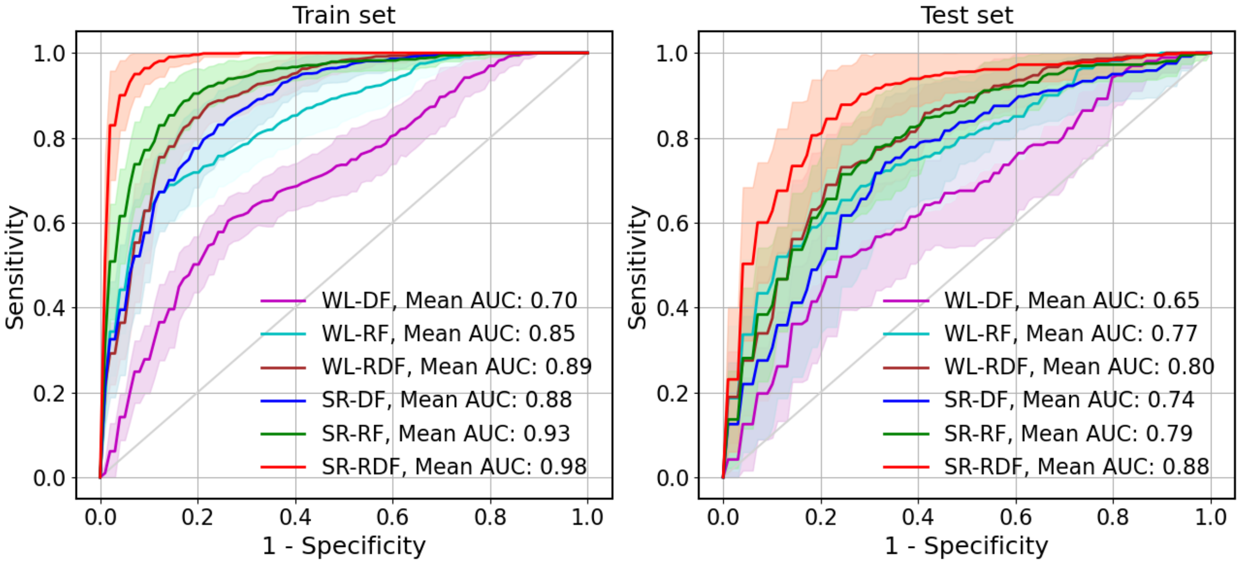

Figure S1. *Mean receiver operating characteristic (ROC) curve of six models using subregion features and the whole lung features.*
